# Tetra­kis(4-meth­oxy­anilinium) hexa­chloridobismuthate(III) chloride monohydrate

**DOI:** 10.1107/S1600536812017096

**Published:** 2012-04-21

**Authors:** Ming-Liang Liu

**Affiliations:** a Ordered Matter Science Research Center, Southeast University, Nanjing 211189, People’s Republic of China

## Abstract

In the crystal of the title compound, (C_7_H_10_NO)_4_[BiCl_6_]Cl·H_2_O, the Bi^III^ cation is located on an inversion center and coordinated by six Cl^−^ anions in a slightly distorted octa­hedral geometry; the uncoordinated Cl^−^ anion and lattice water mol­ecule are located on a twofold rotation axis. Two independent 4-meth­oxy­anilinium cations are linked to the Bi complex, the uncoordinated Cl^−^ anion and lattice water mol­ecule *via* N—H⋯Cl and N—H⋯O hydrogen bonds.

## Related literature

For background literature concerning ferroelectric metal-organic complexes, see: Ye *et al.* (2009 ▶); Zhang *et al.* (2009 ▶, 2010 ▶). For related structures, see: Liu (2011*a* ▶,*b* ▶,*c* ▶).
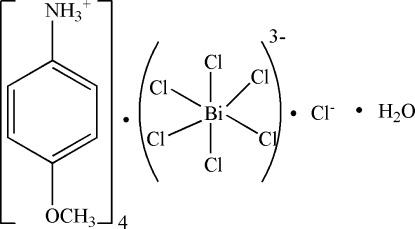


## Experimental

### 

#### Crystal data

(C_7_H_10_NO)_4_[BiCl_6_]Cl·H_2_O
*M* *_r_* = 971.79
Monoclinic, 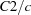

*a* = 25.806 (5) Å
*b* = 7.7081 (15) Å
*c* = 19.550 (4) Å
β = 104.27 (3)°
*V* = 3768.8 (13) Å^3^
*Z* = 4
Mo *K*α radiation
μ = 5.22 mm^−1^
*T* = 293 K
0.21 × 0.20 × 0.20 mm

#### Data collection

Rigaku SCXmini diffractometer
Absorption correction: multi-scan (*CrystalClear*; Rigaku, 2005 ▶) *T*_min_ = 0.350, *T*_max_ = 0.364
18912 measured reflections
4320 independent reflections
3223 reflections with *I* > 2σ(*I*)
*R*_int_ = 0.055

#### Refinement

*R*[*F* ^2^ > 2σ(*F* ^2^)] = 0.031
*wR*(*F* ^2^) = 0.065
*S* = 1.07
4320 reflections
212 parameters
2 restraints
H atoms treated by a mixture of independent and constrained refinement
Δρ_max_ = 0.46 e Å^−3^
Δρ_min_ = −0.89 e Å^−3^

### 

Data collection: *CrystalClear* (Rigaku, 2005 ▶); cell refinement: *CrystalClear*; data reduction: *CrystalClear*; program(s) used to solve structure: *SHELXS97* (Sheldrick, 2008 ▶); program(s) used to refine structure: *SHELXL97* (Sheldrick, 2008 ▶); molecular graphics: *SHELXTL* (Sheldrick, 2008 ▶); software used to prepare material for publication: *SHELXTL*.

## Supplementary Material

Crystal structure: contains datablock(s) I, global. DOI: 10.1107/S1600536812017096/xu5513sup1.cif

Structure factors: contains datablock(s) I. DOI: 10.1107/S1600536812017096/xu5513Isup2.hkl

Additional supplementary materials:  crystallographic information; 3D view; checkCIF report

## Figures and Tables

**Table 1:** Hydrogen-bond geometry (Å, °)

*D*—H⋯*A*	*D*—H	H⋯*A*	*D*⋯*A*	*D*—H⋯*A*
N1—H1*A*⋯O3	0.89	2.01	2.862 (5)	161
N1—H1*B*⋯Cl2	0.89	2.41	3.224 (4)	152
N1—H1*C*⋯Cl1	0.89	2.37	3.231 (4)	165
N2—H2*A*⋯Cl3^i^	0.89	2.67	3.411 (4)	141
N2—H2*A*⋯Cl2^ii^	0.89	2.68	3.330 (4)	131
N2—H2*B*⋯Cl4^iii^	0.89	2.78	3.312 (3)	120
N2—H2*B*⋯Cl3^iv^	0.89	2.83	3.648 (4)	153
N2—H2*C*⋯Cl1^iii^	0.89	2.55	3.418 (4)	167
O3—H3*B*⋯Cl1^v^	0.85 (1)	2.70 (2)	3.202 (6)	119 (2)

Symmetry codes: (i) 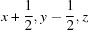; (ii) 
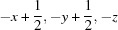
; (iii) 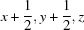; (iv) 
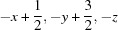
; (v) 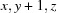.

**Fig. 1.**
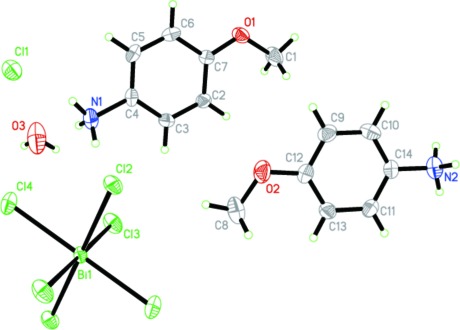
The molecular structure of the title compound, showing the atomic numbering scheme with 30% probability displacement ellipsoids.

**Fig. 2.**
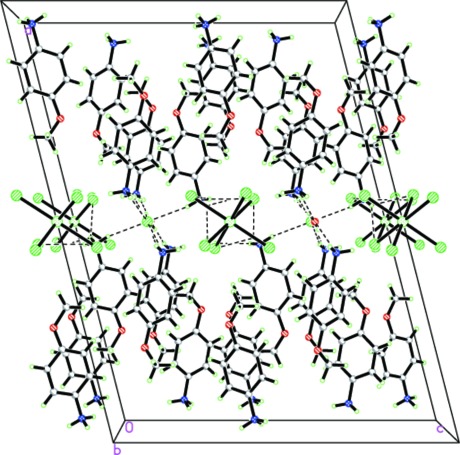
Crystal structure of the title compound with view along the b axis. Intermolecular interactions are shown as dashed lines.

## supplementary crystallographic information

### Comment

Recently much attention has been devoted to simple molecular-ionic compounds containing inorganic and organic ions due to the tunability of their special structural features and their potential ferroelectrics property. Ferroelectric materials that exhibit reversible electric polarization in response to an external electric field have found many applications such as nonvolatile memory storage, electronics and optics. The freezing of a certain functional group at low temperature forces significant orientational motions of the guest molecules and thus induces the formation of the ferroelectric phase. (Zhang *et al.* 2009; Ye *et al.* 2009; Zhang *et al.* 2010). In our laboratory, the title compound has been synthesized and its crystal structure is herein reported.

The title compound, [(C_7_H_10_NO)_4_BiCl_6_]Cl.H_2_O, has an asymmetric unit that consists of two 4-methoxyanilinium cations, half an octahedral hexachloridobismuthate anion, a chloride anion, and half a water molecule (Fig 1). The non-hydrogen atoms of C_7_H_10_NO cations are nearly coplanar, the bismuth atom is coordinated by six chlorides, forming a distorted octahedron, the average Bi—Cl bond distances range from 2.6881 (12) Å to 2.6926 (10) Å, the Cl—Bi—Cl angles range from 85.67 (4)°to 180.00 (7)°. In the crystal, numerous N—H^···^Cl, N—H^···^O, O—H^···^Cl and bifurcated N—H^···^(Cl,Cl) hydrogen bonds link the components to a form layer structure which is parallel to *bc* plane (Fig 2).

### Experimental

4-Methoxylbenzenamine (3.69 g, 0.03 mol) was firstly dissolved in 30 ml ethanol, to which 1.1 g (0.03 mol) of hydrochloric acid was then added to afford the solution, then 3.15 g (0.01 mol) bisumth chloride was dissolved in 20 ml ethanol which was added hydrochloric acid, at last, mixed the above solution without any precipitation under stirring at the ambient temperature. Single crystals suitable for X-ray structure analysis were obtained by the slow evaporation of the above solution after 6 days in air.

The dielectric constant of the compound as a function of temperature indicates that the permittivity is basically temperature-independent (*ε* = C/(T–T_0_)), suggesting that this compound is not ferroelectric or there may be no distinct phase transition occurring within the measured temperature within the measured temperature (below the melting point).

### Refinement

Water H atom was located in a difference Fourier map and refined isotropically. Other H atoms were placed in calculated positions with N—H = 0.89 and C—H = 0.93–0.97 Å, and refined in riding mode with U_iso_(H) = 1.5U_eq_(C,N) for methyl H and amino H atoms, and U_iso_(H) = 1.2U_eq_(C) for the others.

### Crystal data

**Table:**

(C_7_H_10_NO)_4_[BiCl_6_]Cl·H_2_O	*F*(000) = 1920
*M**_r_* = 971.79	*D*_x_ = 1.713 Mg m^−^^3^
Monoclinic, *C*2/*c*	Mo *K*α radiation, λ = 0.71073 Å
Hall symbol: -C 2yc	Cell parameters from 3223 reflections
*a* = 25.806 (5) Å	θ = 0–26°
*b* = 7.7081 (15) Å	µ = 5.22 mm^−^^1^
*c* = 19.550 (4) Å	*T* = 293 K
β = 104.27 (3)°	Block, colourless
*V* = 3768.8 (13) Å^3^	0.21 × 0.20 × 0.20 mm
*Z* = 4	

### Data collection

**Table:**

Rigaku SCXmini diffractometer	4320 independent reflections
Radiation source: fine-focus sealed tube	3223 reflections with *I* > 2σ(*I*)
Graphite monochromator	*R*_int_ = 0.055
CCD_Profile_fitting scans	θ_max_ = 27.5°, θ_min_ = 3.0°
Absorption correction: multi-scan (*CrystalClear*; Rigaku, 2005)	*h* = −33→33
*T*_min_ = 0.350, *T*_max_ = 0.364	*k* = −10→10
18912 measured reflections	*l* = −25→25

### Refinement

**Table:**

Refinement on *F*^2^	Primary atom site location: structure-invariant direct methods
Least-squares matrix: full	Secondary atom site location: difference Fourier map
*R*[*F*^2^ > 2σ(*F*^2^)] = 0.031	Hydrogen site location: inferred from neighbouring sites
*wR*(*F*^2^) = 0.065	H atoms treated by a mixture of independent and constrained refinement
*S* = 1.07	*w* = 1/[σ^2^(*F*_o_^2^) + (0.0215*P*)^2^] where *P* = (*F*_o_^2^ + 2*F*_c_^2^)/3
4320 reflections	(Δ/σ)_max_ < 0.001
212 parameters	Δρ_max_ = 0.46 e Å^−^^3^
2 restraints	Δρ_min_ = −0.89 e Å^−^^3^

### Special details

**Table:**

Geometry. All esds (except the esd in the dihedral angle between two l.s. planes) are estimated using the full covariance matrix. The cell esds are taken into account individually in the estimation of esds in distances, angles and torsion angles; correlations between esds in cell parameters are only used when they are defined by crystal symmetry. An approximate (isotropic) treatment of cell esds is used for estimating esds involving l.s. planes.
Refinement. Refinement of F^2^ against ALL reflections. The weighted R-factor wR and goodness of fit S are based on F^2^, conventional R-factors R are based on F, with F set to zero for negative F^2^. The threshold expression of F^2^ > 2sigma(F^2^) is used only for calculating R-factors(gt) etc. and is not relevant to the choice of reflections for refinement. R-factors based on F^2^ are statistically about twice as large as those based on F, and R- factors based on ALL data will be even larger.

### Fractional atomic coordinates and isotropic or equivalent isotropic displacement parameters (Å^2^)

**Table:**

	*x*	*y*	*z*	*U*_iso_*/*U*_eq_	
O1	0.28804 (11)	0.4079 (3)	0.34243 (14)	0.0518 (7)	
N1	0.07004 (13)	0.4069 (5)	0.21893 (18)	0.0586 (9)	
H1A	0.0550	0.5033	0.2301	0.070*	
H1B	0.0642	0.3995	0.1722	0.070*	
H1C	0.0558	0.3152	0.2350	0.070*	
C1	0.32410 (19)	0.5181 (5)	0.3189 (3)	0.0680 (14)	
H1D	0.3126	0.6364	0.3197	0.102*	
H1E	0.3249	0.4869	0.2716	0.102*	
H1F	0.3592	0.5057	0.3494	0.102*	
C2	0.21434 (17)	0.5117 (4)	0.2491 (2)	0.0421 (9)	
H2	0.2365	0.5776	0.2283	0.050*	
C3	0.16029 (17)	0.5079 (4)	0.2199 (2)	0.0454 (9)	
H3	0.1458	0.5709	0.1790	0.055*	
C4	0.12749 (15)	0.4115 (5)	0.2505 (2)	0.0404 (9)	
C5	0.14777 (17)	0.3185 (5)	0.3107 (2)	0.0482 (10)	
H5	0.1253	0.2536	0.3313	0.058*	
C6	0.20162 (17)	0.3228 (5)	0.33989 (19)	0.0483 (10)	
H6	0.2156	0.2606	0.3811	0.058*	
C7	0.23587 (15)	0.4174 (4)	0.30985 (19)	0.0358 (8)	
O2	0.23658 (13)	0.3700 (4)	0.07934 (16)	0.0683 (9)	
N2	0.44637 (15)	0.5170 (4)	0.0738 (2)	0.0606 (10)	
H2A	0.4616	0.4253	0.0590	0.073*	
H2B	0.4469	0.6062	0.0450	0.073*	
H2C	0.4644	0.5447	0.1173	0.073*	
C8	0.1924 (2)	0.4511 (6)	0.0331 (3)	0.0781 (16)	
H8A	0.1928	0.4259	−0.0149	0.117*	
H8B	0.1945	0.5742	0.0405	0.117*	
H8C	0.1598	0.4081	0.0422	0.117*	
C9	0.32753 (18)	0.3409 (5)	0.1260 (2)	0.0548 (11)	
H9	0.3199	0.2725	0.1614	0.066*	
C10	0.37990 (17)	0.3753 (5)	0.1258 (2)	0.0545 (11)	
H10	0.4075	0.3304	0.1613	0.065*	
C11	0.35099 (19)	0.5412 (5)	0.0212 (2)	0.0500 (11)	
H11	0.3588	0.6095	−0.0141	0.060*	
C12	0.28621 (17)	0.4080 (5)	0.0733 (2)	0.0450 (10)	
C13	0.29834 (18)	0.5055 (4)	0.0210 (2)	0.0500 (10)	
H13	0.2709	0.5483	−0.0153	0.060*	
C14	0.39129 (17)	0.4754 (4)	0.0737 (2)	0.0425 (9)	
Bi1	0.0000	0.5000	0.0000	0.03569 (7)	
Cl2	0.06254 (4)	0.23985 (12)	0.06560 (5)	0.0521 (3)	
Cl3	0.05299 (4)	0.74622 (13)	0.08758 (6)	0.0610 (3)	
Cl4	−0.05859 (5)	0.44282 (15)	0.09398 (6)	0.0623 (3)	
O3	0.0000	0.6668 (7)	0.2500	0.112 (2)	
Cl1	0.0000	0.0822 (2)	0.2500	0.0579 (4)	
H3B	0.0155 (18)	0.741 (2)	0.230 (3)	0.15 (3)*	

### Atomic displacement parameters (Å^2^)

**Table:**

	*U*^11^	*U*^22^	*U*^33^	*U*^12^	*U*^13^	*U*^23^
O1	0.0405 (18)	0.0500 (16)	0.0567 (17)	−0.0059 (14)	−0.0034 (14)	0.0099 (14)
N1	0.038 (2)	0.071 (2)	0.068 (2)	−0.0017 (19)	0.0168 (19)	−0.007 (2)
C1	0.040 (3)	0.066 (3)	0.090 (4)	−0.011 (2)	0.000 (3)	0.011 (2)
C2	0.042 (2)	0.042 (2)	0.042 (2)	−0.0055 (18)	0.0093 (18)	0.0060 (18)
C3	0.043 (3)	0.049 (2)	0.042 (2)	0.0016 (19)	0.0056 (18)	0.0119 (19)
C4	0.032 (2)	0.043 (2)	0.049 (2)	−0.0043 (18)	0.0152 (19)	−0.0112 (19)
C5	0.050 (3)	0.047 (2)	0.055 (3)	−0.0081 (19)	0.027 (2)	0.001 (2)
C6	0.063 (3)	0.047 (2)	0.036 (2)	−0.002 (2)	0.012 (2)	0.0077 (18)
C7	0.039 (2)	0.0319 (19)	0.034 (2)	−0.0023 (17)	0.0050 (18)	−0.0041 (16)
O2	0.047 (2)	0.083 (2)	0.078 (2)	−0.0194 (17)	0.0213 (18)	−0.0023 (18)
N2	0.049 (2)	0.054 (2)	0.081 (3)	−0.0016 (16)	0.021 (2)	−0.0125 (17)
C8	0.045 (3)	0.082 (3)	0.107 (4)	−0.006 (3)	0.018 (3)	−0.021 (3)
C9	0.059 (3)	0.052 (2)	0.055 (3)	−0.010 (2)	0.016 (2)	0.010 (2)
C10	0.054 (3)	0.047 (2)	0.052 (3)	−0.001 (2)	−0.006 (2)	0.006 (2)
C11	0.056 (3)	0.047 (2)	0.053 (3)	0.0013 (19)	0.025 (2)	0.0025 (19)
C12	0.047 (3)	0.040 (2)	0.051 (3)	−0.010 (2)	0.019 (2)	−0.0103 (19)
C13	0.049 (3)	0.054 (2)	0.046 (2)	0.013 (2)	0.010 (2)	0.002 (2)
C14	0.040 (2)	0.034 (2)	0.055 (2)	−0.0028 (17)	0.015 (2)	−0.0067 (18)
Bi1	0.02767 (12)	0.03032 (11)	0.04936 (13)	0.00127 (9)	0.01004 (9)	0.00439 (10)
Cl2	0.0438 (6)	0.0471 (5)	0.0623 (6)	0.0162 (5)	0.0072 (5)	0.0057 (5)
Cl3	0.0500 (7)	0.0438 (6)	0.0786 (8)	−0.0087 (5)	−0.0047 (6)	−0.0071 (5)
Cl4	0.0548 (8)	0.0659 (6)	0.0779 (8)	−0.0033 (5)	0.0387 (6)	0.0005 (6)
O3	0.078 (4)	0.056 (3)	0.217 (7)	0.000	0.063 (5)	0.000
Cl1	0.0523 (10)	0.0516 (8)	0.0652 (10)	0.000	0.0056 (8)	0.000

### Geometric parameters (Å, º)

**Table:**

O1—C7	1.342 (4)	N2—H2B	0.8910
O1—C1	1.418 (5)	N2—H2C	0.8890
N1—C4	1.459 (5)	C8—H8A	0.9600
N1—H1A	0.8902	C8—H8B	0.9600
N1—H1B	0.8898	C8—H8C	0.9600
N1—H1C	0.8890	C9—C10	1.378 (5)
C1—H1D	0.9600	C9—C12	1.387 (5)
C1—H1E	0.9600	C9—H9	0.9300
C1—H1F	0.9600	C10—C14	1.368 (5)
C2—C3	1.371 (5)	C10—H10	0.9300
C2—C7	1.386 (5)	C11—C14	1.365 (6)
C2—H2	0.9300	C11—C13	1.385 (6)
C3—C4	1.370 (5)	C11—H11	0.9300
C3—H3	0.9300	C12—C13	1.367 (5)
C4—C5	1.367 (5)	C13—H13	0.9300
C5—C6	1.367 (5)	Bi1—Cl4^i^	2.6881 (12)
C5—H5	0.9300	Bi1—Cl4	2.6881 (12)
C6—C7	1.383 (5)	Bi1—Cl3	2.6925 (11)
C6—H6	0.9300	Bi1—Cl3^i^	2.6925 (11)
O2—C12	1.347 (4)	Bi1—Cl2	2.6927 (10)
O2—C8	1.414 (5)	Bi1—Cl2^i^	2.6927 (10)
N2—C14	1.456 (5)	O3—H3B	0.846 (10)
N2—H2A	0.8910		
			
C7—O1—C1	118.4 (3)	O2—C8—H8B	109.5
C4—N1—H1A	109.5	H8A—C8—H8B	109.5
C4—N1—H1B	109.4	O2—C8—H8C	109.5
H1A—N1—H1B	109.4	H8A—C8—H8C	109.5
C4—N1—H1C	109.6	H8B—C8—H8C	109.5
H1A—N1—H1C	109.5	C10—C9—C12	120.1 (4)
H1B—N1—H1C	109.4	C10—C9—H9	120.0
O1—C1—H1D	109.5	C12—C9—H9	120.0
O1—C1—H1E	109.5	C14—C10—C9	120.1 (4)
H1D—C1—H1E	109.5	C14—C10—H10	119.9
O1—C1—H1F	109.5	C9—C10—H10	119.9
H1D—C1—H1F	109.5	C14—C11—C13	119.6 (4)
H1E—C1—H1F	109.5	C14—C11—H11	120.2
C3—C2—C7	119.9 (4)	C13—C11—H11	120.2
C3—C2—H2	120.1	O2—C12—C13	125.7 (4)
C7—C2—H2	120.1	O2—C12—C9	115.3 (4)
C4—C3—C2	120.3 (4)	C13—C12—C9	119.0 (4)
C4—C3—H3	119.9	C12—C13—C11	120.8 (4)
C2—C3—H3	119.9	C12—C13—H13	119.6
C5—C4—C3	120.9 (4)	C11—C13—H13	119.6
C5—C4—N1	119.0 (4)	C11—C14—C10	120.4 (4)
C3—C4—N1	120.1 (4)	C11—C14—N2	118.8 (4)
C6—C5—C4	118.8 (4)	C10—C14—N2	120.8 (4)
C6—C5—H5	120.6	Cl4^i^—Bi1—Cl4	180.00 (3)
C4—C5—H5	120.6	Cl4^i^—Bi1—Cl3	92.06 (4)
C5—C6—C7	121.7 (4)	Cl4—Bi1—Cl3	87.94 (4)
C5—C6—H6	119.1	Cl4^i^—Bi1—Cl3^i^	87.94 (4)
C7—C6—H6	119.1	Cl4—Bi1—Cl3^i^	92.06 (4)
O1—C7—C6	116.2 (3)	Cl3—Bi1—Cl3^i^	180.00 (7)
O1—C7—C2	125.4 (3)	Cl4^i^—Bi1—Cl2	94.33 (4)
C6—C7—C2	118.5 (4)	Cl4—Bi1—Cl2	85.67 (4)
C12—O2—C8	118.8 (4)	Cl3—Bi1—Cl2	94.08 (4)
C14—N2—H2A	109.5	Cl3^i^—Bi1—Cl2	85.92 (4)
C14—N2—H2B	109.6	Cl4^i^—Bi1—Cl2^i^	85.67 (4)
H2A—N2—H2B	109.3	Cl4—Bi1—Cl2^i^	94.33 (4)
C14—N2—H2C	109.6	Cl3—Bi1—Cl2^i^	85.92 (4)
H2A—N2—H2C	109.4	Cl3^i^—Bi1—Cl2^i^	94.08 (4)
H2B—N2—H2C	109.5	Cl2—Bi1—Cl2^i^	180.00 (6)
O2—C8—H8A	109.5		
			
C7—C2—C3—C4	0.2 (5)	C12—C9—C10—C14	−0.2 (6)
C2—C3—C4—C5	0.4 (6)	C8—O2—C12—C13	−7.6 (6)
C2—C3—C4—N1	−179.6 (3)	C8—O2—C12—C9	172.2 (4)
C3—C4—C5—C6	−0.2 (6)	C10—C9—C12—O2	−178.5 (3)
N1—C4—C5—C6	179.8 (3)	C10—C9—C12—C13	1.2 (6)
C4—C5—C6—C7	−0.5 (6)	O2—C12—C13—C11	177.9 (4)
C1—O1—C7—C6	−172.6 (4)	C9—C12—C13—C11	−1.8 (6)
C1—O1—C7—C2	8.1 (5)	C14—C11—C13—C12	1.3 (6)
C5—C6—C7—O1	−178.4 (3)	C13—C11—C14—C10	−0.2 (6)
C5—C6—C7—C2	1.0 (5)	C13—C11—C14—N2	−178.9 (3)
C3—C2—C7—O1	178.5 (3)	C9—C10—C14—C11	−0.3 (6)
C3—C2—C7—C6	−0.8 (5)	C9—C10—C14—N2	178.3 (3)

Symmetry code: (i) −*x*, −*y*+1, −*z*.

### Hydrogen-bond geometry (Å, º)

**Table:**

*D*—H···*A*	*D*—H	H···*A*	*D*···*A*	*D*—H···*A*
N1—H1*A*···O3	0.89	2.01	2.862 (5)	161
N1—H1*B*···Cl2	0.89	2.41	3.224 (4)	152
N1—H1*C*···Cl1	0.89	2.37	3.231 (4)	165
N2—H2*A*···Cl3^ii^	0.89	2.67	3.411 (4)	141
N2—H2*A*···Cl2^iii^	0.89	2.68	3.330 (4)	131
N2—H2*B*···Cl4^iv^	0.89	2.78	3.312 (3)	120
N2—H2*B*···Cl3^v^	0.89	2.83	3.648 (4)	153
N2—H2*C*···Cl1^iv^	0.89	2.55	3.418 (4)	167
O3—H3*B*···Cl1^vi^	0.85 (1)	2.70 (2)	3.202 (6)	119 (2)

Symmetry codes: (ii) *x*+1/2, *y*−1/2, *z*; (iii) −*x*+1/2, −*y*+1/2, −*z*; (iv) *x*+1/2, *y*+1/2, *z*; (v) −*x*+1/2, −*y*+3/2, −*z*; (vi) *x*, *y*+1, *z*.
